# The Dual Impact of Pretest Sensitisation and the Cognitive Acceleration Through Science Education Programme in the Solomon Four-Group Design

**DOI:** 10.3390/brainsci16010064

**Published:** 2025-12-31

**Authors:** Mourad El Karkri, Antonio Quesada, Marta Romero-Ariza

**Affiliations:** Department of Didactics of Science, University of Jaén, 23071 Jaén, Spain; antquesa@ujaen.es (A.Q.); mromero@ujaen.es (M.R.-A.)

**Keywords:** cognitive acceleration, pretest sensitisation, Solomon four-group design, educational research, statistical analysis

## Abstract

**Background/Objectives:** Many studies have investigated the Cognitive Acceleration through Science Education (CASE) programme, demonstrating its impact on students’ reasoning and learning development across different educational contexts. Likewise, numerous experimental investigations have employed the Solomon Four-Group Design (SFGD) to control for pretest sensitisation and improve the validity of intervention studies. However, despite the extensive use of both frameworks independently, no previous research has integrated them within a single study. The present research therefore combines the theoretical foundations of CASE with the methodological rigour of the SFGD to explore the influence of intervention outcomes under different pretest conditions on learners’ cognitive growth. **Methods:** This study examines differences associated with pretest sensitisation and the CASE programme among middle school students using a quasi-experimental research design. The study was conducted with 88 students divided into four groups, two experimental and two control, following the Solomon Four-Group Design to account for pretest sensitisation and its potential interaction with the treatment. **Results:** Statistical analyses revealed that the observed outcomes differed between pretested and non-pretested groups, with the pretested conditions showing larger post-test differences than the non-pretested ones. **Conclusions:** Rather than establishing causal effects, this study highlights key methodological considerations related to pretest sensitisation when evaluating cognitive acceleration interventions. The findings provide practical guidance for researchers and educators in designing, analysing, and interpreting classroom-based intervention studies where pretesting may influence observed outcomes.

## 1. Introduction

Recent findings in the study of educational neuroscience have resulted in a general consensus that there is significance in knowing how the brain learns, as it enables us to construct effective practices aiming to achieve instruction and cognitive results [1]. The determination of associations between neuroscience literacy and improved learning behaviour and school outcomes indicates that the ability of learners to conduct metacognitive regulation and reasoning depends on a basic awareness of key neurocognitive mechanisms [1]. Such relevance emerges because CASE is grounded in psychological principles of cognitive development that resonate with key assumptions in educational neuroscience. This perspective is complementary to the intent and aims of the Cognitive Acceleration through Science Education programme (CASE). The programme was initially designed for students aged 12–14, replacing one science lesson every two weeks over two years in order to develop reasoning skills in topics such as variables, proportionality, probability, and formal modelling [2,3]. It comprises 30 lessons structured to enhance cognitive development through activities fostering critical thinking and problem-solving [4].

Evaluated using a quasi-experimental design, the programme demonstrated significant cognitive gains, with participating students often outperforming peers by one grade level [4]. Its effects, particularly in areas such as scientific creativity, have been reported to show enduring impacts rather than immediate results [5]. Adaptations for other disciplines, such as mathematics, have further highlighted improvements in self-regulation, motivation, and academic achievement [6].

Widely implemented across Europe [7,8,9], Australia [10,11,12,13], USA [14,15], Asia [3,16,17,18], and Africa [19,20], CASE has demonstrated its effectiveness in diverse socio-cultural settings and subjects. Its units are based on five key principles of the “Thinking Science” approach: (1) concrete preparation, (2) cognitive conflict, (3) social construction, (4) metacognition, and (5) bridging [2]. Teachers play a pivotal role in guiding discussions, fostering cognitive conflict, and supporting reasoning development throughout the process. Despite the extensive evidence supporting the effectiveness of CASE across diverse contexts, relatively little attention has been paid to how assessment-related factors, such as pretest sensitisation, may interact with CASE interventions and influence the interpretation of their effects.

Building on these structured approaches to developing students’ reasoning, broader pedagogical frameworks have emphasised engaging learners in activities that promote exploration and conceptual reconstruction, aligning with the logic underlying inquiry-based methodologies. Inquiry-based learning (IBL) has attracted considerable attention, particularly following its promotion in the United States in the late 20th and early 21st centuries and later in Europe through influential expert reports [21,22]. As an alternative to traditional direct instruction, IBL has been praised for fostering critical thinking [23] and problem-solving skills [24].

However, some researchers argue that IBL has been overemphasised, overshadowing the role of direct teaching methods. Disparities in findings, including negative associations, have been reported in some studies [25,26], underlining the need to examine the conditions under which IBL is most effective. Investigations over the last decade, often through meta-analyses, have explored factors influencing the effectiveness of IBL. For instance, Spronken-Smith et al. [27] highlighted the importance of institutional support, teacher characteristics, and effective lesson design. They also emphasised the use of thought-provoking questions to promote reasoning development, which aligns with key principles of the CASE approach.

Evidence syntheses have increasingly focused on the conditions that maximise the effectiveness of IBL. Lazonder and Harmsen [28], reviewing 72 studies, underscored the importance of structured guidance, which aligns with the CASE model in which teachers actively guide reasoning and challenge students’ ideas during experimentation. Romero-Ariza et al. [24] further emphasised that effective IBL incorporates opportunities for argumentation and modelling, enabling students to evaluate evidence and construct explanatory models in line with the three-dimensional view of science learning [29] and Osborne’s perspectives [30]. Consistent with this, Öztürk et al. [31] reviewed meta-analyses published between 2015 and 2022 and highlighted the effectiveness of inquiry-based approaches, particularly modelling, in developing critical thinking and inquiry skills, with benefits for scientific literacy and conceptual understanding [32].

The CASE approach exemplifies high-quality IBL, engaging students in investigating physical phenomena, challenging their ideas, and refining them through experimental evidence. This study aims to evaluate the impact of a CASE-based intervention on students’ reasoning skills, particularly their ability to control variables.

Based on the background outlined above, this study applies a Solomon four-group quasi-experimental research design to address to the following research questions (RQ):

1. RQ1: How does the implementation of a series of five CASE programme lessons affect students’ reasoning skills, particularly their ability to control variables, compared to students who did not receive the programme and relative to their reasoning skills before the intervention?
2. RQ2: Does pretest sensitisation (PS) occur in this study, and how does it influence students’ outcomes compared with those who did not receive the pretest?

## 2. Materials and Methods

### 2.1. Materials

This study was conducted within the framework of extracurricular activities organised by the “Scientific and Technological Club”, an initiative established by a secondary school teacher in Morocco.

The study was carried out between the last week of April and the end of May 2022, offering students an opportunity to engage in structured scientific learning outside their regular classroom environment. It was based on a sequence of five CASE lessons, selected from the original set of 30 developed by Adey et al. [2], representing a significantly shorter version of the full programme. Each lesson included one, two, or three activities designed to be completed within an hour.

Certain lessons need the preparation of particular resources for the hands-on phase. In this intervention, the following materials were prepared (see Table 1) and please see the Supplementary Materials for other results:

### 2.2. Method

For each activity, note sheets were prepared and shared with groups of four to five students to promote collaboration during their investigations and to help them record observations and findings. In addition, work cards were developed that included images or real-world scenarios, allowing students to connect the type of thinking fostered in the lessons with practical situations during the bridging phase.

The intervention was implemented by the club’s teacher, who also contributed to the research. To mitigate potential bias associated with this dual role, the implementation was standardised across groups using the same lesson sequence, materials (note sheets and work cards), and session duration. The pre- and post-tests were administered under uniform conditions, and scoring followed pre-defined criteria aligned with the assessment instrument. In addition, the results and interpretations are presented cautiously in light of the quasi-experimental context.

The lesson sequence started with a class discussion around the question “What do scientists do?” Students offered a range of responses such as conducting experiments, performing research, making precise measurements, and observing with microscopes. The teacher then spent a few minutes organising these ideas to highlight that one of the main goals of science is to discover and understand relationships among different phenomena. To reinforce this point, the teacher linked the discussion to previous lessons. For instance, in the “Earth’s Atmosphere” unit, students had observed that temperature decreases as altitude increases. In the given case, both temperature and altitude were treated as variables, each capable of taking two values: high and low. By analysing these earlier examples, students were guided to recognise the difference between values and variables.

**Figure 1:**
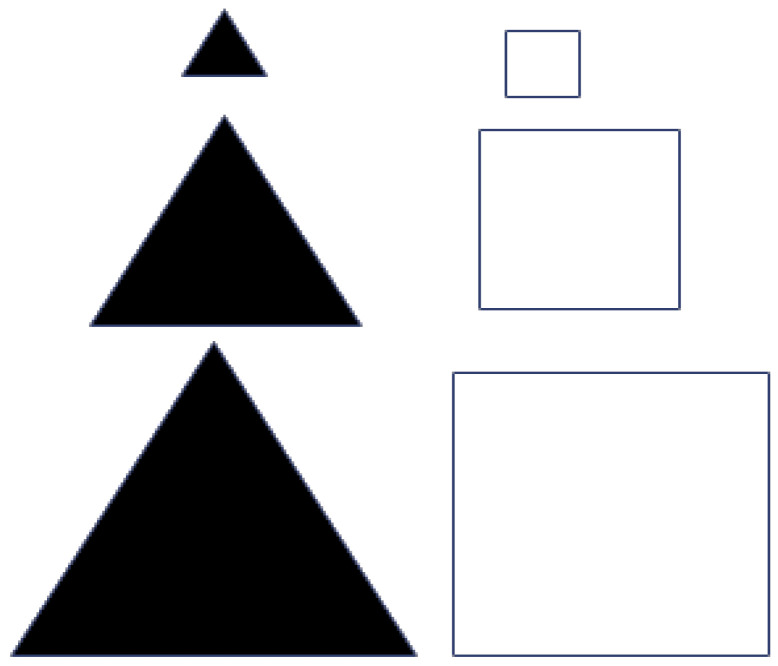
(Set 1) Variations in laminated shapes: white and black, triangles and squares, and small, medium, and large sizes.

Among all the activities outlined in Table 1, activity 1 “What Varies?” is presented here in detail as an example, while the remaining activities were also implemented separately, each during a two-hour session. The first activity began by showing students a set of laminated cardboard shapes (see Figure 1) and asking them “What are the differences between these shapes?” Through examining the shapes, students identified three key variables: colour (two possible values: white and black), shape (two possible values: triangle and square), and size (three possible values: small, medium, and large). They also observed that shapes sharing the same form had the same colour, which illustrated a relationship between two variables—shape and colour. The activity concluded the “concrete preparation” phase by introducing and familiarising students with the terms “variable”, “value”, and “relationship”.

**Figure 2:**
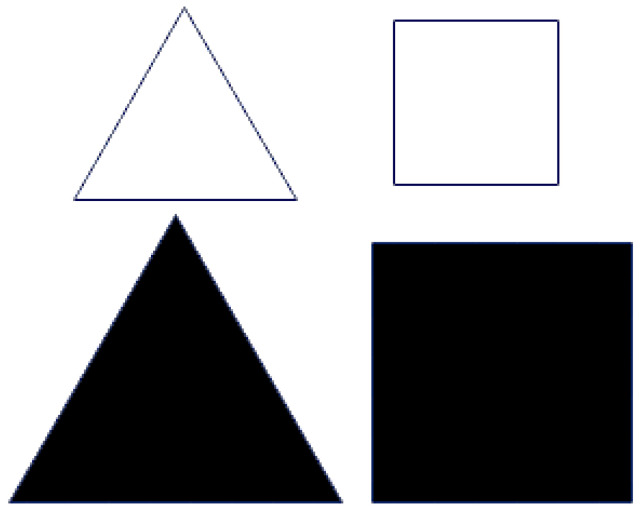
(Set 2) Introducing a cognitive conflict to explore whether there is a relationship between variables and values.

Set 2 (see Figure 2) was created to trigger “cognitive conflict” by challenging students’ underlying assumptions about consistent relationships among variables such as colour, shape, and size. Although secondary school students usually recognise that objects can vary in form, they often develop habitual expectations based on past experiences—for instance, linking specific shapes with certain colours. The objects in Set 2 were intentionally chosen to disrupt these expectations by presenting unexpected or contradictory combinations. Unlike Set 1, where size, colour, and shape followed clear, structured patterns, Set 2 deliberately broke or altered these relationships. This inconsistency was designed to provoke students to question and reconsider their earlier assumptions.

To support students’ reasoning and reflection, the following guiding questions were posed:

- What are the variables and their possible values?
- Does a relationship still exist between colour and shape?
- Are any of the variables connected to one another?
- If so, in which way are they related?

These prompts encouraged students to revisit and reconstruct their thinking, examining whether the causal or categorical patterns they previously expected were still valid. In doing so, the questions strengthened the cognitive conflict process at the heart of the activity.

The cognitive conflict phase involved working closely and thinking critically. In Set 2, students examined their patterns. They were asked to rethink their ideas about connections between shape, colour, and size. With the teacher’s help, they refined their thinking about variability so that their understanding became clearer. This stage of learning involved a constant exchange between integrating new ideas and modifying old ones, resulting in a cycle of mutual construction of meaning. To keep the discussion going, the teacher regularly prompted students’ thinking by asking guiding questions such as “If we put a black square on the right, what might we put on the left?”

The metacognition phase encouraged students to reflect on their own thinking processes by examining their responses and evaluating their validity through logical reasoning and evidence-based discussion. The teacher supported this stage by posing reflective prompts such as “Could you explain the reasons behind your choice? Why or why not?”, which guided students to articulate, justify, and critically assess their thought processes.

The bridging phase aimed to transfer the cognitive skills developed in the CASE-based lessons—particularly recognising variables and analysing their relationships—into wider areas of the science curriculum and daily life. To strengthen this reasoning approach, the teacher intentionally incorporated it into later topics, such as experimental design in physics and data analysis in biology. This continuity enabled students to extend their analytical thinking beyond the initial lessons and appreciate its importance in authentic scientific investigation.

### 2.3. Research Design

This study employs the Solomon Four-Group Design (SFGD), which is one of the most robust frameworks for experimental research [33,34]. The structure of this design is presented in Table 2. The SFGD is particularly effective in controlling for PS and testing the interaction effects between pretesting and the treatment effect. It is considered a highly rigorous design for ensuring reliable and valid results, as detailed by El Karkri et al. [35].

The SFGD plays a pivotal role in the accurate evaluation of educational programmes by supporting precise assessments and guiding the future development of such initiatives. By adopting this design, researchers are better equipped to address challenges such as PS and to measure intervention effects with greater accuracy. This not only ensures the reliability of assessments, but also contributes significantly to the advancement of educational research across various domains [36,37,38,39,40].

### 2.4. Sample and Context

The middle school where the study was conducted is situated in a rural zone near Tangier in the north of Morocco. This institution serves students from nearby villages, reflecting the characteristics of educational institutions in such areas. The college where the research was conducted was selected due to its accessibility and the first author’s role as a teacher at the institution. He is a physics and chemistry teacher there, though not directly responsible for the participating students. This professional connection facilitated the coordination of research activities and provided an informed understanding of the local educational context.

Eighty-eight (88) middle school students participated in the study as a conventional sample coming from four natural classes within a quasi-experimental research design. The four classes belonged to the same school, shared a common socio-economic background, and were taught by the same teacher. These classes, each comprising students aged 13 to 14 years, were officially established as part of the school’s regular structure. For the purposes of the Solomon Four-Group Design (SFGD), each class was randomly assigned to one of the four experimental groups: two experimental groups and two control groups. Thus, in this study, each Solomon “group” corresponded to one intact “class”, reflecting both the design requirements and the organisational reality of the school. The four-group research design allows for a systematic comparison among the four groups, enabling us to estimate the effect size of the intervention considering the possibility of pretest sensitisation (See Table 3).

However, differences in group sizes emerged due to irregular attendance across the five scheduled classes, as well as during the pre- and post-tests. This variability in attendance was influenced by the schooling conditions in a semi-rural environment, coupled with family-related factors and transportation challenges. Consequently, the study only considered students who attended at least three of the five scheduled classes. Thus, for the pre- and post-tests of the control groups, the number of participants was larger, as these tests required minimal time and were conducted in a single session.

Although each class initially included 40 students according to the school regulations, participation in the study was strictly voluntary after having been informed of the purpose and conditions, causing differences in group sizes. Among those who agreed to participate on a previously informed and voluntary basis, there were no withdrawal cases. However, some students were unable to attend all the sessions scheduled for the intervention due to personal or family reasons. In Group 1, six students participated in the programme but were absent on the day of the post-test for personal reasons and were thus excluded from the final analysis. If they had attended the day on which the post-test was administered, the first group would have contained 15 rather than 9 students.

### 2.5. Instruments and Measures

The structure of the questions was based on problem-solving scenarios inspired by previous research [41], while the content was directly taken from CASE programme materials. The validity of item content is supported by the alignment of the questions used with those proposed by the CASE programme. Those questions were designed to unveil students’ reasoning skills and have been successfully applied as a valid instrument in previous research studies [35]. Both the pretest and post-test consisted of multiple-choice questions, with a maximum score of 15 points for correctly answering all items. Participants from both the control and experimental groups were given one hour to complete each test.

Data were gathered through the students’ worksheets questionnaires by summing the total score of each test, where each question required marking an ‘X’ on the correct option and selecting the correct reasoning for that choice. Test scores were then saved into IBM SPSS Statistics (Version 26.0), including different variables that may be used in the analysis: post-test score, pretest score, and gain score (the difference between the post-test and the pretest).

**Figure 3:**
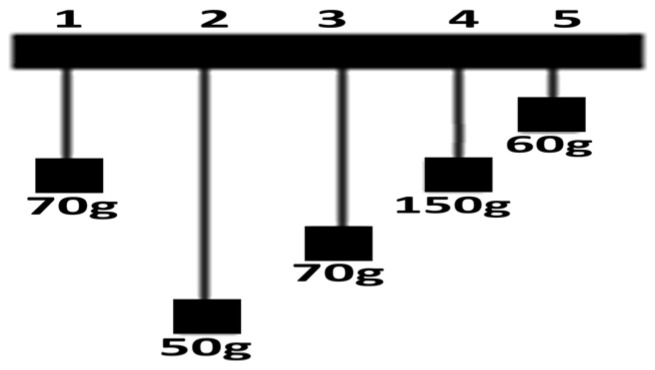
Five pendulums with different masses used in a sample experimental question.

The following question represents a sample item included in the instrument:

“Imagine we want to do an experiment to see if changing the weight of a pendulum changes how long it takes to swing from one side to the other. Among the five pendulums shown in the Figure 3, which pendulums should we use to carry out this kind of experiment?”

□ 1 and 4.

□ 2 and 4

□ 1 and 3

□ 2 and 5

□ All the pendulums

Select the correct justification for your answer:

□ We should compare a lighter pendulum with a heavier one.

□ We should compare all the pendulums with each other.

□ The pendulums selected should have the same length and differ only in mass.

□ The pendulums selected should have the same mass and differ only in length.

For scoring purposes, each correct response to the main question was awarded 0.5 points, and an additional 0.5 points were assigned for selecting the correct justification. The items in the instrument differed in punctuation and structure: some consisted of a single question with one justification, while others contained multiple sub-questions or more than one justification option that required careful reading. Similar items followed this general format, including images of various situations. Examples are outlined as follows: a closed syringe to explore how the volume of enclosed air varies with different applied forces on the plunger; a set of different balls used to determine which ball bounces the highest under comparable conditions; variation in fountain height h as a function of distance d, corresponding to the initial height of the water inside the funnel.

## 3. Results

### 3.1. Correlation Between Post-Test and Pretest Scores

To explore how students’ early performance influenced later outcomes, a linear regression analysis was carried out. In this analysis, pretest scores were used to predict post-test scores for both Group 1 and Group 2, following the method described by Odom & Morrow Jr. [42]. As shown in Table 4, pretest scores explained 49.4% of the variation in post-test scores (R^2^ = 0.494; Adjusted R^2^ = 0.478), suggesting a moderate to strong relationship between the two. The correlation coefficient (R = 0.703) also supports this connection between students’ initial and subsequent performance

A detailed examination of the regression coefficients (Table 5) confirms that pretest scores are a significant predictor of post-test scores (B = 0.671, β = 0.703, t = 5.588, *p* < 0.001). This finding indicates that an increase in pretest scores corresponds to an increase in post-test scores.

These findings offer early evidence of score stability across the two test administrations.

### 3.2. Reliability and Internal Consistency of Instrument

To evaluate the internal consistency of the measurement instrument, Guttman’s Lambda reliability coefficients were computed. These coefficients provide a refined measure of reliability by assessing different methods of partitioning the test items [43]. The results, presented in Table 6, indicate the extent to which the instrument maintains consistency across its components.

The values for λ_1_, λ_2_, λ_3_, λ_4_, and λ_5_ demonstrate a strong level of internal reliability, confirming the consistency of the measurement instrument. λ_1_ (0.762), which represents the most basic measure of internal consistency, indicates a solid foundation for reliability. The values for λ_2_, λ_3_, λ_4_, and λ_5_ all exceed 0.80, suggesting that the test items are well aligned and consistently measure the intended construct. Additionally, the highest reliability coefficient, λ_5_, accounts for unequal item variances, further reinforcing the robustness of the instrument. These findings provide strong empirical support for the reliability of the instrument, confirming its suitability for measuring the targeted constructs. The high internal consistency suggests that the instrument is well structured and produces stable measurements, ensuring confidence in its application for research purposes. The λ_6_ coefficient was not computed by SPSS due to limitations in the covariance matrix, which resulted in a system-missing value for this statistic.

Internal consistency was also examined using Cronbach’s alpha, which indicated good reliability (α = 0.802) for the 20-item instrument, consistent with the Guttman’s Lambda coefficients.

### 3.3. Data Normality

Before proceeding with the statistical analyses, it is necessary to verify the normality of the data to ensure the appropriateness of the chosen methods. Assessing normality provides a foundation for determining whether parametric tests can be applied or if non-parametric alternatives are required.

The Kolmogorov–Smirnov test indicates no significant deviations from normality for outcomes O1, O2, O4, and O6, with *p* ≥ 0.067. However, O5 displays a significant result (*p* = 0.040), suggesting a potential departure from normality in this outcome. The Shapiro–Wilk test, which is more sensitive for smaller sample sizes, corroborates these findings. All outcomes exhibit *p*-values up to 0.05 (0.926 (O1), 0.839 (O2), 0.664 (O3), 0.351 (O4), 0.198 (O5), and 0.274 (O6)), indicating that the null hypothesis of normality cannot be rejected. This includes O5, where the Shapiro–Wilk test suggests no significant deviation (*p* = 0.198) despite the Kolmogorov–Smirnov result. In summary, while the Kolmogorov–Smirnov test highlights a potential departure from normality for O5, the Shapiro–Wilk test, which is often preferred for small sample sizes, suggests that the data for all outcomes, including O5, are normally distributed. These findings support the use of parametric statistical methods for further analysis, provided other assumptions are met.

Beyond verifying the normality of the data, it is important to consider the potential impact of outliers, particularly given the small size of some of our experimental groups, where even minor deviations can disproportionately influence the results.

Huber’s M-Estimator, Tukey’s Biweight, Hampel’s M-Estimator, and Andrews’ Wave were applied to the given outcomes (O1 to O6). These robust estimators are specifically designed to mitigate the influence of outliers [44], providing more reliable measures of central tendency compared to analysis through central tendency means, particularly in datasets that may deviate from normality.

A notable observation is the general consistency of values across the different M-Estimators for each category, with only minor variations evident between methods. For instance, for O5, the M-Estimator values range from 8.8706 (Huber’s M-Estimator) to 8.9333 (Hampel’s M-Estimator), reflecting a small variability of just 0.0627. This suggests that the data are relatively stable and not heavily influenced by extreme values or significant outliers. Similarly, O2 consistently exhibits the highest values across all estimators, ranging from 11.0849 (Hampel’s M-Estimator) to 11.1876 (Andrews’ Wave), while O4 presents some of the lowest values, with estimates ranging from 7.8077 (Huber’s M-Estimator) to 7.8894 (Hampel’s M-Estimator). Such differences could indicate variations in central tendencies among these groups, which may warrant further investigation into their potential causes.

The range of values across all categories extends from 7.8077 (O4, Huber’s M-Estimator) to 11.1876 (O2, Andrews’ Wave), demonstrating a good level of robustness. The minor variations observed between methods suggest that the dataset is relatively stable and not significantly affected by extreme values or outliers. This robustness ensures that the central tendency measures are reliable and provides a solid foundation for further analysis and group comparisons.

### 3.4. Descriptive Statistics

Descriptive statistics for the pretest scores were first examined to understand initial performance levels. A comparison between the two groups shows a higher average post-test performance for the Experimental & Pretested group (M = 9.61) compared to the Control & Pretested group (M = 7.86). The variability in scores was similar across groups, with standard deviations of 2.67 and 2.72, respectively. Table 7 presents the descriptive statistics.

The Experimental & Pretested group had a notably higher mean pretest score than the Control & Pretested group, M = 9.61 versus M = 7.86, with confidence intervals suggesting that there was likely to be a significant difference at baseline. The range of scores for the experimental group was also somewhat smaller, 5.50 to 13.50, compared to the 1.00 to 12.50 range for the control group, although the amount of variation measured by the standard deviation was similar for both. These descriptive differences suggest that students in the experimental group entered the study with generally higher initial performance and reinforce why baseline variation must be considered when interpreting post-test outcomes for this design.

Descriptive statistics for the post-test results of the four Solomon groups are given in Table 8.

The highest mean post-test score was reached by the “Experimental & Pretested” group, with *n* = 9 (M = 10.94, SD = 2.26, 95% CI [9.21, 12.68]), followed by the “Control & Non-pretested” group, with *n* = 37 (M = 9.35, SD = 2.11, 95% CI [8.65, 10.06]), and then the “Experimental & Non-pretested” group, with *n* = 17 (M = 8.94, SD = 1.98, 95% CI [7.92, 9.96]). The lowest mean score after the test was observed in the control group with pretest conditions, (*n* = 25; SD = 2.29, 95% CI [6.85, 8.75]) at 7.80. These descriptive results at the group level give a clearer basis for the subsequent analysis of treatment, pretest, and sensitization effects within the Solomon design. The observed minimum and maximum values suggest heterogeneous baseline performance across students in both groups, underscoring the importance of considering initial variability when interpreting subsequent post-test differences.

These descriptive patterns provide an initial overview of how the four Solomon groups performed on the post-test and highlight significant differences between the pretested and non-pretested conditions.

Although the four groups represented different intact classes, all students be-longed to the same school, were taught by the same teacher, and worked in the same educational and comparable environment. This minimises the likelihood of differences between the groups arising from class distinctions. Nevertheless, class affiliation remained synonymous with group affiliation, a methodological limitation that must be noted, as the experiment was designed using pre-existing, naturalistic classes rather than randomly assigning students to individual classes.

### 3.5. Analysis of Pretest Sensitisation

The primary objective of the SFGD is to detect whether an interaction exists between the pretest and the treatment. To achieve this, a Two-Way Analysis of Variance (ANOVA) was conducted. This test assesses the interaction effect between the pretest and the treatment on the dependent variable, post-test scores, aiming to determine whether exposure to the pretest influenced the treatment’s measurement, thereby identifying potential PS effects.

The results of Levene’s test indicate that the assumption of homogeneity of variances was met (F(3.84) = 0.299, *p* = 0.826). In the Two-Way ANOVA, the main effect of the treatment revealed a statistically significant effect (F(1.84) = 6.794, *p* = 0.011), indicating that the treatment contributed significantly to the variance in post-test scores. The main effect of the pretest was not statistically significant (F(1.84) = 0.186, *p* = 0.668), suggesting that the pretest alone did not significantly influence the post-test scores. However, the interaction between the treatment and the pretest was statistically significant (F(1.84) = 11.482, *p* = 0.001). The F-value indicates the proportion of variance in the dependent variable explained by the interaction relative to the unexplained variance, and the associated *p*-value confirms that this interaction effect is statistically significant. This result suggests that the combination of the pretest and the treatment had a measurable and meaningful impact on the dependent variable.

### 3.6. Managing Pretest Sensitisation

The detection of a significant interaction between the pretest and the treatment underscores the necessity of looking at the pretested and non-pretested groups as distinct categories in subsequent analyses. This approach enables a more precise examination of the treatment’s effects within each group and ensures that the influence of the pretest is adequately considered. Such an analysis allows for the identification of one of several possible scenarios: the treatment may exert a significant effect in both groups; the magnitude or direction of the treatment effect may differ between the pretested and non-pretested groups; or the treatment may prove effective in only one of the two groups. Additionally, although less likely given the significant main effect of the treatment, it is possible for the treatment’s effectiveness within each subgroup to be individually non-significant, while the interaction effect alone drives the observed significance. Differentiating between these scenarios is essential for achieving a nuanced understanding of the interaction between the pretest and the treatment, as well as for drawing valid and robust conclusions about the overall effectiveness of the treatment.

### 3.7. Analysis of Post-Test

The first parametric test conducted was an independent-samples *t*-test to compare the post-test scores (O2 and O4) of the pretested groups. This comparison examined whether post-test performance differed significantly between participants who received the treatment (O2) and those who did not (O4) under the pretested condition. Levene’s test for equality of variances confirmed that the assumption of homogeneity of variances was met (F = 0.131, *p* = 0.720).

The *t*-test showed a statistically significant difference in post-test scores between the treated group (O2) and the untreated group (O4), with a corresponding *p*-value of 0.001. The mean difference between the two groups was 3.144, with a 95% confidence interval ranging from 1.337 to 4.951. These results indicate a statistically significant difference in post-test scores between the treated and untreated groups under the pretested condition, favouring the treated group.

The second parametric test compared the post-test scores of the non-pretested groups, Group 3 and Group 4 (O5 Compared to O6). Levene’s test confirmed homogeneity of variances (F = 0.680, *p* = 0.413), allowing a *t*-test assuming equal variances to be performed. The independent-samples *t*-test revealed no statistically significant difference in post-test scores between the treated and untreated groups within the non-pretested condition (*p* = 0.502).

The statistical analysis showed a statistically significant difference in the pretested groups between the treated and untreated conditions. In contrast, in the non-pretested groups, no significant difference emerged between the treated and untreated conditions, which might be considered an indicator of PS.

### 3.8. Effect Size

To estimate the magnitude of the differences between groups, standardised mean differences (Cohen’s d) were calculated using the pooled standard deviation, following the formula $d=\frac{M_{1}-M_{2}}{SD_{\mathrm{pooled}}}$, where $SD_{\mathrm{pooled}}=\sqrt{\frac{(n_{1}-1)SD_{1}^{2}+(n_{2}-1)SD_{2}^{2}}{n_{1}+n_{2}-2}}$. Using this approach, the comparison between the pretested treatment group (*n* = 9, M = 10.94, SD = 2.26) and the pretested control group (*n* = 25, M = 7.80, SD = 2.29) yielded a large effect size (d = 1.38), indicating a substantial standardised difference on the post-test. In contrast, the comparison between the non-pretested treatment group (*n* = 17, M = 8.94, SD = 1.98) and the non-pretested control group (*n* = 37, M = 9.35, SD = 2.11) produced a small and negligible effect size (d = –0.20), slightly favouring the control group. These results might be interpreted as a combined effect of both the pretest and the treatment on students’ reasoning skills, thus suggesting again the need to check potential PS.

## 4. Discussion

The results show that the pretested groups exhibited higher post-test scores, suggesting a possible interaction between the pretest and subsequent performance and the need to consider potential PS through a rigorous research design such as the Solomon’s four-group research design.

The independent-samples *t*-test was conducted to compare the post-test scores of Group 1 (O2: pretested and treated) and Group 3 (O5: treated but non-pretested). Levene’s test for equality of variances indicated that the assumption of equal variances was met (F = 0.770, *p* = 0.389). The results showed a statistically significant difference between the two groups (t(24) = 2.338, *p* = 0.028), with a mean difference of 2.003 (95% CI: 0.235 to 3.771). This finding suggests that the pretested and treated group (Group 1) scored significantly higher on the post-test compared to the treated but non-pretested group (Group 3), a result that aligns with previous findings.

Similarly, a comparison between the post-test scores of Group 1 (O2: pretested and treated) and Group 4 (O6: untreated and non-pretested) was conducted. The results, while approaching significance (t(44) = −2.005, *p* = 0.051), indicate a notable difference in performance, with Group 1 scoring higher than Group 4.

The latest test examined whether the pretest alone influenced behaviour independently of the treatment. A *t*-test was conducted to compare the post-test scores of the pretested and untreated group, Group 2 (O4), with the non-pretested and untreated group, Group 4 (O6). The independent-samples *t*-test revealed a statistically significant difference (t(60) = −2.743, *p* = 0.008), with equal variances assumed (*p* = 0.901). These findings suggest that the act of pretesting itself may have influenced participants’ performance, underscoring the potential impact of PS on post-test outcomes.

Beyond the statistical differences observed between the experimental groups, the findings may also be interpreted in terms of underlying cognitive processes. The observed PS effect may be understood as reflecting attentional activation and heightened task awareness induced by initial exposure to assessment items. Pretesting can orient learners’ attention towards relevant variables and reasoning demands, thereby priming subsequent engagement with instructional content [45]. In this sense, PS can be viewed as a reactive cognitive process rather than a purely methodological artefact, consistent with meta-analytic evidence on sensitisation effects [46,47].

In addition to these cognitive considerations, the rural educational context may also have shaped the pretest sensitisation effect. In many educational systems, rural schooling contexts often overlap with lower socioeconomic conditions, which may increase the salience of assessment-based activities. This interpretation is consistent with research showing that contextual disadvantage can influence students’ engagement with cognitive acceleration interventions in low-SES schools [48].

These results also invite a brief reflection on classroom practice. Pretesting may serve as a brief “orientation” that alerts students to the reasoning demands of upcoming CASE activities. Teachers might therefore use a small set of low-stakes diagnostic items at the start of a lesson to elicit initial thinking and draw attention to the relevant variables. Where the aim is to estimate instructional impact without reactive testing effects, alternative options include delaying formal assessment, using parallel forms, or explicitly presenting the pretest as diagnostic rather than evaluative. In each case, a short follow-up discussion can help channel any sensitisation into learning rather than confounding interpretation.

## 5. Conclusions

This study may be the first one to explore the potential of CASE while considering the possibility of PS through the SFGD. While these findings are limited to the present sample, they suggest that the pretest experience influences students’ performance in the post-test, particularly when combined with the treatment, as the highest post-test scores were observed in the group subjected to both the pretest and the treatment. In addition, quasi-experimental research comparing different groups requires the application of appropriate statistical methods, such as ANCOVA, to control for baseline differences, together with robust measures to support reliable results.

In this particular case, the influence of the pretest on students’ later performance might be due to the nature of the items included. As previously explained, items in the pretest and post-test are highly aligned with the CASE approach and have been designed to collect evidence of students’ reasoning skills. To this end, items in the tests are based on scenarios that provoke and guide students’ thinking, prompting them to provide an appropriate response. Additionally, exposure to these questions in the pretest could enhance students’ motivation to explore the content of the CASE sessions. In contrast, students who enter the sessions directly, without this prior engagement, may show lower levels of attention than those who took the pretest.

While the absence of PS allows for a straightforward execution of the treatment process described by Braver & Braver [49], its presence introduces complexity that requires additional analytical considerations. Previous research conducted by El Karkri et al. [35] shows how the existence, or lack thereof, of PS depends on the particular circumstances under study and might be explained considering the nature of the tests applied. Nevertheless, the possibility of PS should be considered in order to avoid misattributions.

Future research should further explore these dynamics across diverse contexts and samples, including educational settings with varying geographical characteristics, such as rural contexts, to build a more comprehensive understanding of pretest and treatment interactions.

## 6. Limitations

The present study did not include item-level analyses such as item difficulty, discrimination indices, or item–total correlations. Additionally, neither alternate-form nor test–retest procedures were implemented. These aspects will be addressed in future research to further strengthen construct validity and the psychometric robustness of the assessment instrument.

One methodological limitation of this study is the small sample size and the fact that the distribution of students among the four groups reflects natural class grouping and therefore a quasi-experimental research design. The four groups were not perfectly balanced in size, which may reduce statistical power for some comparisons and widen confidence intervals, increasing the risk of Type II error. Accordingly, non-significant findings should be interpreted cautiously and not taken as evidence of no effect. To mitigate this limitation, analyses were conducted using appropriate group comparison procedures with explicit assumption checks, and the interpretation emphasises the pattern of results across the Solomon four-group structure rather than reliance on isolated *p*-values. A further line of work would be to replicate the study using a random distribution and with larger samples of students.

Finally, the pre- and post-test questions were built around the content of the CASE lessons, which may have made pretested students more familiar with the type of questions used during the intervention to stimulate reasoning skills and the ones used later on to evaluate these skills after the intervention. Therefore, it is necessary to reflect on the nature of the items used for pre- and post-testing and its potential influence on students’ final performance.

## Figures and Tables

**Table 1:** The resources and materials required for the CASE-based intervention following the first five lessons (control of variables).

Lessons	Activities and Equipment Required
What varies?	Coloured shapes: Square and triangular shapes, white and black, and in three different sizes (see Figure 1 and Figure 2). Four Boxes: Four boxes A, B, C, and D—Pan Balance.
Two variables	3. The pulley: Dynamometer—Mass hanger—Three identical masses (100 g)—Method of fixing string to base. 4. Heights of liquid: One 100 cm^3^ beaker—Four circular containers all at least 100 cm^3^ in volume of varying heights and diameters—Coloured Water—Ruler. 5. Drying leaves: Leaves from trees—Vaseline jelly.
What sort of relationship?	6. Stretching a spring: spring and fixing support—Ruler—5 masses. 7. Heating oil: Cancelled due to a lack of safety and protection measures.
The fair test	8. What affects the note: Pipes (or bars) of two different materials (wood, iron, glass, plastic…), different lengths (long, medium, short), and different widths (wide, medium, and narrow).
Roller ball	9. Roller ball: Track on which balls can roll (one end steep and one end flat)—Balls of different materials and sizes.

**Table 2:** The Solomon Four-Group Design.

Groups	Assignment	Pretest	Treatment	Post-Test
Experimental 1	Random	Yes (O1)	Yes	Yes (O2)
Control 1	Random	Yes (O3)	No	Yes (O4)
Experimental 2	Random		Yes	Yes (O5)
Control 2	Random		No	Yes (O6)

Code. O1 through O6 represent different outcomes of the results.

**Table 3:** The Solomon Four-Group Design and sample size for each group.

Groups	Assignment	Size Group	Group Percentage	Pretest	Treatment	Post-Test
Experimental 1	Random	9	10.2%	O1	Yes	O2
Control 1	Random	25	28.4%	O3	No	O4
Experimental 2	Random	17	19.3%		Yes	O5
Control 2	Random	37	42.0%		No	O6

**Table 4:** Model summary of regression analysis examining predictive relationship between pretest and post-test scores.

Model	R	R Square	Adjusted R Square	Std. Error of the Estimate
1	0.703 ^a^	0.494	0.478	1.91628

^a^ Predictors: (Constant), pretest score.

**Table 5:** Regression coefficients estimating predictive influence of pretest scores on post-test performance.

Coefficients ^a^								
Model		Unstandardized Coefficients		Standardised Coefficients	t	Sig.	95.0% Confidence Interval for B	
		B	Std. Error	Beta			Lower Bound	Upper Bound
1	(Constant)	3.045	1.053		2.893	0.007	0.901	5.189
	Pretest	0.671	0.120	0.703	5.588	0.000	0.427	0.916

^a^ Dependent Variable: post-test.

**Table 6:** Guttman’s Lambda reliability coefficients for assessing internal consistency of measurement instrument.

Lambda	1	0.762
	2	0.837
	3	0.802
	4	0.825
	5	0.838
	6	-
N of Items		20

**Table 7:** Means, standard deviations, and confidence intervals for pretest scores.

Solomon Group	N	Mean	SD	95% CI for Mean	Min	Max
Experimental (Pretested)	9	9.61	2.67	[7.56, 11.66]	5.50	13.50
Control (Pretested)	25	7.86	2.72	[6.74, 8.98]	1.00	12.50

**Table 8:** Means, standard deviations, and confidence intervals for post-test scores.

Solomon Group	N	Mean	SD	95% CI for Mean	Min	Max
Experimental (Pretested)	9	10.94	2.26	[9.21, 12.68]	7.00	14.00
Control (Pretested)	25	7.80	2.29	[6.85, 8.75]	2.50	12.00
Experimental (Non-pretestedted)	17	8.94	1.98	[7.92, 9.96]	4.00	12.50
Control (Non-pretested)	37	9.35	2.11	[8.65, 10.06]	5.00	13.00

## Data Availability

The data supporting the findings of this study are not publicly available due to privacy restrictions. The datasets consist of anonymized student responses collected for research purposes only and are available from the corresponding author upon reasonable request.

## Supplementary Materials

The following supporting information can be downloaded at: https://www.mdpi.com/article/10.3390/brainsci16010064/s1, Table S1: Results of Normality Tests for Data Distribution Across Categories (O1 to O6): O1 and O3 Represent Pre-test Scores, while O2, O4, O5, and O6 Represent Post-test Scores (as Described in Table 1 in the manuscript). Table S2: Analysis of Data Using M-Estimators for Categories (O1 to O6): Assessing Robustness and Reliability for Group Comparisons. Table S3: Levene’s Test for Equality of Error Variances for Post-Test Scores (Test of Homogeneity). Table S4: Two-Way ANOVA Test: Analysis of Between-Subjects Effects for Treatment, Pre-test, and Their Interaction on Post-test Scores. Table S5: *t*-test analysis of Post-test variable (Pretested Groups 1 and 2). Table S6: *t*-Test Analysis of Post-Test Scores in Non-Pretested Groups 3 (O5) and 4 (O6). Table S7: Levene’s Test for Equality of Error Variances for Post-Test Scores. Table S8: ANCOVA: Tests of Between-Subjects Effects on Post-Test Scores. Table S9: Independent *t*-Test Comparing Post-Test Scores of Pretested Untreated Group 2 (O4) and Non-Pretested Untreated Group 4 (O6). Table S10: Correlation Between Pre-Test and Post-Test Scores in Pretested Untreated Group 2 (O3 vs. O4). Table S11: Paired Samples *t*-test Comparing Pre-test and Post-test Scores in Pretested Untreated Group 2 (O3 vs. O4). Table S12: Independent Samples *t*-test Comparing Pre-Test Scores (O3) and Post-Test Scores (O6). Table S13: Correlation Between Pre-Test (O1) and Post-Test (O2) Scores. Figure S1: Correlation Between Pre-Test and Post-Test Scores in Pretested Untreated Group 2.

## Abbreviations

The following abbreviations are used in this manuscript:

CASE	Cognitive Acceleration through Science Education
SFGD	Solomon Four-Group Design
IBL	Inquiry-Based Learning
PS	Pretest sensitisation
ANCOVA	Analysis of Covariance
ANOVA	Analysis of Variance
