# Supplementary material for: The Dual Impact of Pretest Sensitisation and the Cognitive Acceleration Through Science Education Programme in the Solomon Four-Group Design
Source: Brain Sci. 2025 Dec 31;16(1):64. doi: 10.3390/brainsci16010064 (PMC12839333; doi:10.3390/brainsci16010064)
Supplement: Supplementary file 1 [file brainsci-16-00064-s001.zip › brainsci-4066424-supplementary.pdf]

These are the statistical results obtained from SPSS. We found that it was not necessary to include the classical tables of standard tests in the main document. Instead, we retained only the table related to the Solomon design and the tables presenting the validity and reliability tests.

Table S1:Results of Normality Tests for Data Distribution Across Categories ($O1$ to $O6$): $O1$ and $O3$ Represent Pre-test Scores, while $O2$, $O4$, $O5$, and $O6$ Represent Post-test Scores (as Described in Table 1 in the manuscript).

	Kolmogorov-Smirnov[a]			Shapiro-Wilk		
Oi	Statistic	df	Sig.	Statistic	df	Sig.
O1	0.114	9	0.200*	0.974	9	0.926
O2	0.153	9	0.200*	0.964	9	0.839
O3	0.108	25	0.200*	0.971	25	0.664
O4	0.168	25	0.067	0.957	25	0.351
O5	0.213	17	0.040	0.927	17	0.198
O6	0.107	37	0.200*	0.964	37	0.274

*. This is a lower bound of the true significance.

a. Lilliefors Significance Correction

Table S2: Analysis of Data Using M-Estimators for Categories ($O1$ to $O6$): Assessing Robustness and Reliability for Group Comparisons.

	M-Estimators			
Oi	Huber's M-Estimator[a]	Tukey's Biweight[b]	Hampel's M-Estimator[c]	Andrews' Wave[d]
O1	9.7139	9.6688	9.6460	9.6684
O2	11.1701	11.1901	11.0849	11.1876
O3	7.8827	7.9773	7.9230	7.9785
O4	7.8077	7.8784	7.8894	7.8734
O5	8.8706	8.9179	8.9333	8.9187
O6	9.3827	9.4082	9.4097	9.4069

a. The weighting constant is 1,339.

b. The weighting constant is 4,685.

c. The weighting constants are 1,700, 3,400, and 8,500

d. The weighting constant is 1,340*pi.

Table S3: Levene’s Test for Equality of Error Variances for Post-Test Scores (Test of Homogeneity).

Levene's Test of Equality of Error Variances[a]

Dependent Variable: POSTTEST

F	df1	df2	Sig.
0.299	3	84	0.826

Tests the null hypothesis that the error variance of the dependent variable is equal across groups.

a. Design: Intercept + Treatment + PreTested + Treatment * PreTested

Table S4: Two-Way ANOVA Test: Analysis of Between-Subjects Effects for Treatment, Pre-test, and Their Interaction on Post-test Scores.

Dependent Variable: POSTTEST

Source	Type III Sum of Squares	df	Mean Square	F	Sig.
Corrected Model	74.651[a]	3	24.884	5.358	0.002
Intercept	5788.857	1	5788.857	1246.525	0.000
Treatment	31.550	1	31.550	6.794	0.011
PreTested	0.862	1	0.862	0.186	0.668
Treatment * PreTested	53.322	1	53.322	11.482	0.001
Error	390.096	84	4.644		
Total	7583.750	88			
Corrected Total	464.747	87			

a. R Squared = 0.161 (Adjusted R Squared = 0.131)

Table S5: T-test analysis of Post-test variable (Pretested Groups 1 and 2).

Independent Samples Test

		Levene's Test for Equality of Variances		t-test for Equality of Means						
		F	Sig.	t	df	Sig. (2-tailed)	Mean Difference	Std. Error Difference	95% Confidence Interval of the Difference Lower	95% Confidence Interval of the Difference Upper
POSTTEST	Equal variances assumed	0.131	0.720	3.544	32	0.001	3.14444	0.88730	1.33708	4.95181
	Equal variances not assumed			3.570	14.383	0.003	3.14444	0.88067	1.26029	5.02860

Table S6: T-Test Analysis of Post-Test Scores in Non-Pretested Groups 3 (O5) and 4 (O6).

Independent Samples Test

		Levene's Test for Equality of Variances		t-test for Equality of Means						
		F	Sig.	t	df	Sig. (2-tailed)	Mean Difference	Std. Error Difference	95% Confidence Interval of the Difference	
									Lower	Upper
POST-TEST	Equal variances assumed	0.680	0.413	-0.675	52	0.502	-0.41017	0.60728	-1.62876	0.80841
	Equal variances not assumed			-0.692	33.015	0.494	-0.41017	0.59317	-1.61696	0.79661

Table S7: Levene's Test for Equality of Error Variances for Post-Test Scores.

Levene's Test of Equality of Error Variances[a]

Dependent Variable: POSTTEST

F	df1	df2	Sig.
0.007	1	32	0.935

Tests the null hypothesis that the error variance of the dependent variable is equal across groups.

a. Design: Intercept + PRETEST + FourSolomonGroups

Table S8: ANCOVA: Tests of Between-Subjects Effects on Post-Test Scores.

Tests of Between-Subjects Effects

Dependent Variable: POSTTEST

Source	Type III Sum of Squares	df	Mean Square	F	Sig.	Partial Eta Squared
Corrected Model	142.526[a]	2	71.263	24.648	0.000	0.614
Intercept	52.263	1	52.263	18.076	0.000	0.368
PRETEST	77.094	1	77.094	26.665	0.000	0.462
FourSolomonGroups	27.880	1	27.880	9.643	0.004	0.237
Error	89.628	31	2.891			
Total	2765.750	34				
Corrected Total	232.154	33				

a. R Squared = 0.614 (Adjusted R Squared = 0.589)

Table S9: Independent T-Test Comparing Post-Test Scores of Pretested Untreated Group 2 (O4) and Non-Pretested Untreated Group 4 (O6).

Independent Samples Test

		Levene's Test for Equality of Variances		t-test for Equality of Means						
		F	Sig.	t	df	Sig. (2-tailed)	Mean Difference	Std. Error Difference	95% Confidence Interval of the Difference	
									Lower	Upper
POSTTEST	Equal variances assumed	0.016	0.901	-2.743	60	0.008	-1.55135	0.56567	-2.68285	-0.41985
	Equal variances not assumed			-2.699	48.737	0.010	-1.55135	0.57484	-2.70670	-0.39600

Table S10: Correlation Between Pre-Test and Post-Test Scores in Pretested Untreated Group 2 (O3 vs O4).

Paired Samples Correlations

		N	Correlation	Sig.
Pair 1	Pretest_G2 & Posttest_G2	25	0.681	0.000

Table S11: Paired Samples T-test Comparing Pre-test and Post-test Scores in Pretested Untreated Group 2 (O3 vs O4).

Paired Samples Test

		Paired Differences					t	df	Sig. (2-tailed)
		Mean	Std. Deviation	Std. Error Mean	95% Confidence Interval of the Difference				
					Lower	Upper			
Pair 1	Pretest_G2 - Posttest_G2	0.06000	2.03777	0.40755	-0.78115	0.90115	0.147	24	0.884

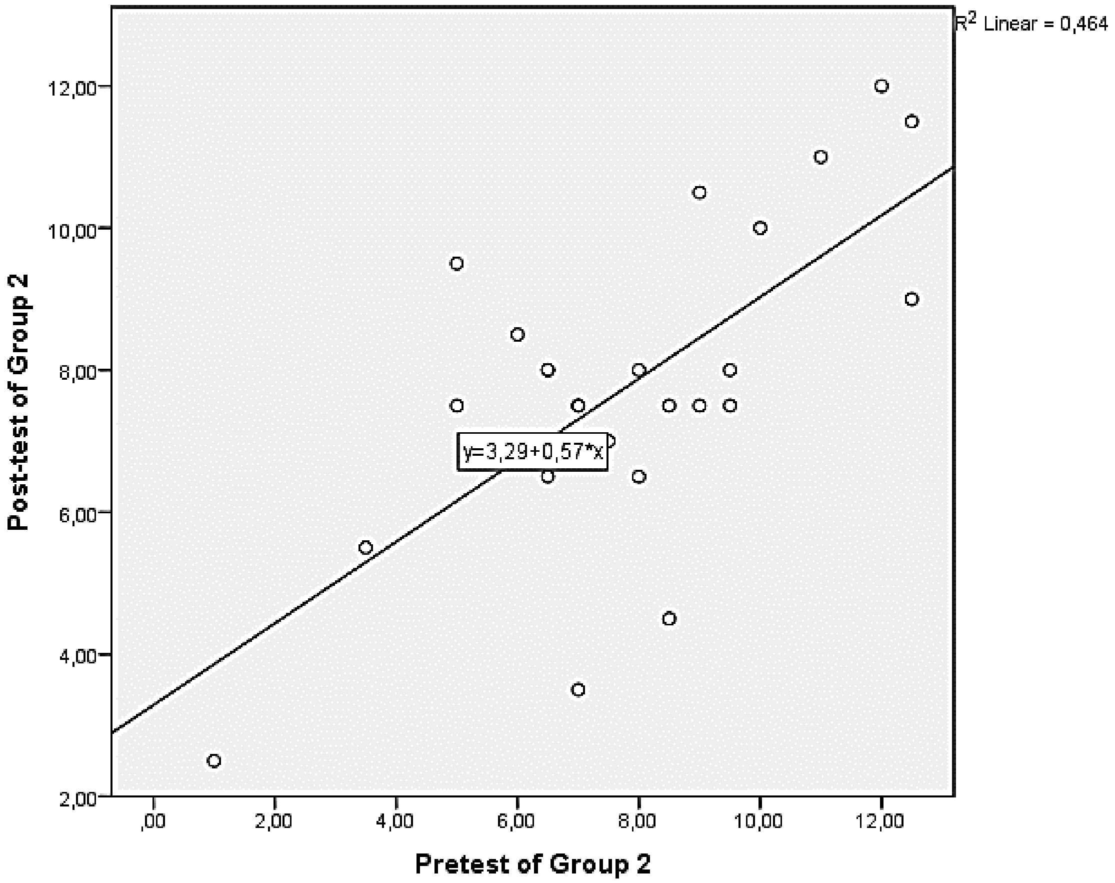

Figure S1: Correlation Between Pre-Test and Post-Test Scores in Pretested Untreated Group 2.

Table S12: Independent Samples T-test Comparing Pre-Test Scores (O3) and Post-Test Scores (O6).

Independent Samples Test

		Levene's Test for Equality of Variances		t-test for Equality of Means						
		F	Sig.	t	df	Sig. (2-tailed)	Mean Difference	Std. Error Difference	95% Confidence Interval of the Difference	
									Lower	Upper
O3_vs_O6	Equal variances assumed	1.064	0.306	-2.428	60	0.018	-1.49135	0.61420	-2.71993	-0.26277
	Equal variances not assumed			-2.313	42.814	0.026	-1.49135	0.64489	-2.79205	-0.19065

Table S13: Correlation Between Pre-Test (O1) and Post-Test (O2) Scores.

Paired Samples Correlations				
		N	Correlation	Sig.
Pair 1	O1 & O2	9	0.676	0.045
